# Potential of Subcritical Water Hydrolysis to Valorize Low-Valued Ray-Finned Fish (*Labeobarbus nedgia*): Effects of Hydrolysis Temperature and Pressurization Agent

**DOI:** 10.3390/foods13101462

**Published:** 2024-05-09

**Authors:** Solomon Abebaw Tadesse, Shimelis Admassu Emire, Pedro Barea, Alba Ester Illera, Rodrigo Melgosa, Sagrario Beltrán, María Teresa Sanz

**Affiliations:** 1 Department of Food Engineering, School of Chemical and Bioengineering, Addis Ababa Institute of Technology, Addis Ababa University, Addis Ababa P.O. Box 385, Ethiopia; solomon.abebaw@aastu.edu.et (S.A.T.); shimelis.admassu@aait.edu.et (S.A.E.); 2 Department of Food Science and Applied Nutrition, College of Applied Sciences, Addis Ababa Science and Technology University, Addis Ababa P.O. Box 16417, Ethiopia; 3 Chemical Engineering Section, Department of Biotechnology and Food Science, University of Burgos, 09001 Burgos, Spain; pbgomez@ubu.es (P.B.); aeillera@ubu.es (A.E.I.); rmgomez@ubu.es (R.M.); beltran@ubu.es (S.B.)

**Keywords:** low-valued ray-finned fish, subcritical water hydrolysis, nitrogen, carbon dioxide, degree of hydrolysis, pressurization agent

## Abstract

Subcritical water (SCW) hydrolysis was applied to valorize the low-valued ray-finned fish (*Labeobarbus nedgia*) into valuable protein hydrolysates, employing N_2_ and CO_2_ as pressurization agents at varying temperatures (140, 160, 180, and 200 °C). The degree of hydrolysis (DH) and total free amino acid content increased with temperature for both pressurizing agents. The highest DH (54.5 ± 0.4%) and total free amino acid content (210 ± 1 mg/g_prot_) were observed at 200 °C when CO_2_ gas was used as the pressurizing agent. Predominantly, glycine and alanine were released for both pressurizing agents. The antioxidant activity, evaluated through three different assays, increased with temperature and was found to be the highest at 200 °C. This study illustrated the advantages of the intensified SCW technology by using CO_2_ as a pressurization agent in valorizing low-valued ray-finned fish (*Labeobarbus nedgia*), as animal residue rich in proteins, for the production of valuable protein hydrolysates with a high fraction of valuable free amino acids, which could offer potential applications as a functional ingredient in the food industry.

## 1. Introduction

In recent years, the utilization of low-valued fish and fish by-products has gained significant attention in the field of food science, nutrition, medicine, and biotechnology. These underutilized resources possess a high potential for the production of valuable bioactive compounds such as antioxidant peptides and free amino acids [1]. 

On a global scale, about 38.5 million tons of different types of fish are discarded as by-catch due to their low commercial value [2], even though they are actually a great source of protein. *Labeobarbus* spp. are endemic fish to Africa and constitute a family of about 80 large cyprinid fish species, which are widely distributed in the large rivers in Africa such as Nile, Niger, Congo and Zambezi and in the Great Rift and other Lakes of East Africa, south to KwaZulu-Natal in the east and the Orange and Clan William Olifants Rivers in the west [3]. However, these fish species are low-valued because of the presence of small bones inside the meat that make them difficult for consumption, and thus unacceptable to consumers in the market [4], which consider these species by-catch fish. Therefore, taking these considerations into account, *Labeobarbus* fish species could be utilized for the production of bioactive peptides and free amino acids helping to create new value chains from by-catch fish species contributing to fish waste valorization.

Hydrolysis converts higher molecular weight proteins into lower molecular weight peptides, which have gained a great interest in the food, pharmaceutical and cosmetic industries due to their several bio-functional properties such as antioxidant and antimicrobial [5]. Enzymatic and chemical hydrolysis have been widely employed in the hydrolysis of proteins. Although enzymes have a wide application in the hydrolysis of proteins, limitations such as high-cost of enzymes, potential allergenicity, and time-consuming processes have prompted researchers to explore alternative hydrolysis methods [1]. On the other hand, chemical hydrolysis methods involve the use of strong acids or alkalis, which can pose risks to human health because of toxic residues they may leave in the final products that may result in the degradation of bioactive compounds [6]. Moreover, peptides obtained from chemical hydrolysis contain a large amount of salt, as a result of the pH neutralization [7]. Hence, there is a need for the development of safer and more efficient hydrolysis techniques for the production of bioactive peptides and free amino acids from low-valued fish [2]. 

Subcritical water hydrolysis, also known as hot pressurized water extraction, offers a promising alternative to conventional hydrolysis methods. Subcritical water (SCW) is water in its liquid state in the range of 100 to 374 °C and with pressures up to 22 MPa [8]. Under these conditions, water presents unique properties, such as the decrease in viscosity, density, and dielectric constant, facilitating the solubilization of non-polar components [9]. The concentration of ionic products, hydronium (H_3_O^+^) and hydroxide (OH^−^), also increase, which facilitates water to act as an acid- or base-like catalyst for hydrolysis reactions [10]. Thus, SCW, which combines high pressure and high temperature, has been recognized as a green technology to convert protein from diverse sources into bioactive peptides and free amino acids [11]. However, high temperature and long treatment time result in the degradation of amino acids to organic acids [5]. Therefore, appropriate conditions, especially a combination of time and temperature, are very important to produce proper functional materials.

Different types of gases have been used to generate the required pressure level in SCW systems [12]. Among the possible gases, nitrogen is the most widely used gas due to its nonreactive nature. However, other reactive gases such as carbon dioxide have been reported as a significant factor influencing the physicochemical properties of the hydrolysate because such gases are considered to act as a catalyzers or modifiers [13]. Using carbon dioxide as a pressurizing agent in the SCW system leads to a more acidic medium, due to the formation of carbonic acid that serves as a catalyst, thus enhancing the hydrolysis of proteins [14,15]. Barea et al. [10] also reported that the addition of carbon dioxide led to an increase in amino acid production from tuna fish meal compared to nitrogen. Therefore, the choice of pressurizing gases in SCW treatment is also important. 

Recently, SCW technology has been employed in the hydrolysis of proteins from different fish and fish wastes [9,10]. However, there are no previous studies on the valorization of *Labeobarbus nedgia* using SCW process to produce biofunctional materials. Therefore, the aim of this study was to evaluate the effects of hydrolysis temperature and pressurizing agents on the free amino acid profile, antioxidant activity and physicochemical properties of protein hydrolysates from *Labeobarbus nedgia* by SCW hydrolysis. 

## 2. Materials and Methods

### 2.1. Materials

*Labeobarbus nedgia* (LB), sourced from Lake Tana in Ethiopia, was caught and utilized as a protein source. The fish were collected during the morning hours and promptly transported to the Food and Chemical Engineering laboratory at Bahir Dar University in ice boxes. Upon arrival, the fish were promptly filleted, and the muscle was rinsed twice with freshwater. Subsequently, the muscle was freeze-dried by transferring the muscle, that was pre-frozen at a temperature below −30 °C, into a vacuum chamber of the freeze-drier (Mini Lyodel, Chennai, India). Finally, the dried muscle was ground, packaged in polyethylene bags, and stored at −20 °C for future use.

### 2.2. Proximate Composition Analysis

The AOAC International’s Standard Methods [16] were employed to assess the moisture, crude fat, and ash contents in LB muscle. The protein content was determined using the total nitrogen content obtained through elemental analysis applying the corresponding N-factor derived from the amino acid profile of the LB muscle protein.

### 2.3. Elemental Analysis

The LB muscle’s elemental composition (including C, H, N, S) was analyzed using the Flash 2000 elemental microanalyzer (Thermo Scientific, Waltham, MA, USA). The oxygen content was determined through mass balance. 

### 2.4. Amino Acid Profile Analysis

The amino acid composition of the LB muscle was evaluated using a gas chromatograph (Hewlett-Packard, 6890 series, PA, USA) equipped with an EZ:faast AAA LC integrated column and FID detector, as outlined by Alonso-Riaño et al. [17]. LB muscle (1 g) was subjected to hydrolysis through mixing with 1 mL of 6 M HCl, followed by incubation at 110 °C for 24 h. Following this, 1 mL of a 1 M HCl and ethanol solution (1:1 *v*/*v*) was mixed, and the mixture was filtered using a 0.2 μm cellulose acetate syringe filter. It is worth noting that during acid hydrolysis, asparagine and glutamine are completely converted into aspartic and glutamic acid, respectively. Additionally, tryptophan, cystine, and cysteine may be degraded by acid hydrolysis, and methionine could be partially degraded as well. To address this, basic hydrolysis was employed to analyze these amino acids. This involved mixing 0.1 g of LB muscle with 7 mL of 4.2 M NaOH, followed by a 24 h incubation at 110 °C. The mixture was then cooled, and its pH was adjusted to between 1.5 and 5.5 by neutralizing it with 6 M HCl. After hydrolysis, the amino acid profile was determined using the EZ:faast Phenomenex procedure described by Trigueros et al. [18], which includes a solid phase extraction, followed by derivatization, and a final liquid/liquid extraction step.

### 2.5. Equipment for Subcritical Water Hydrolysis

Subcritical water hydrolysis of LB was carried out in a lab assembled batch system with a reactor of 0.5 L capacity. The reactor was covered by a ceramic heating jacket (230 V, 4000 W, ø 95 mm, 160 mm height) to reach the selected working temperature. A Pt100 sensor placed inside the reactor and the PID system, to which it is connected, allowed us to control and register the temperature during the hydrolysis. A needle valve (Autoclave Engineers, PA, USA), followed by a cooling system, was connected to collect samples along the SCW treatment.

In a typical run, a homogenized mixture of 200 mL distilled water and 20 g of the LB muscle were charged into the reactor (10 wt%). Four different temperatures, 140, 160, 180, and 200 °C, and two pressurizing agents, N_2_ or CO_2_ gases, at a working pressure of 50 bar, were assayed. The working pressure was selected based on previous studies of the research group for biomass valorization from different sources [10,17,18]. The effect of pressure on the performance of hydrolysis in different studies has been observed as non-significant, compared with temperature and time, as long as water remains in the liquid state [13].

Hydrolysis kinetics were followed by carefully withdrawing samples at regular time intervals through the sampling port. About 2 mL of hydrolyzed samples were collected every 10 min for the first 60 min of hydrolysis, followed by 30 min intervals for the remainder of the hydrolysis process. The time zero was considered when the operating temperature was reached. After 240 min, the vessel was cooled and then depressurized when the temperature was lower than 90 °C.

### 2.6. Degree of Hydrolysis, Protein Content, and Total Hydrolysis Yield

The degree of hydrolysis (DH) was determined using the ninhydrin reaction method outlined in the Sigma Aldrich (St. Louis, MI, USA) protocol. Specifically, 1 mL of ninhydrin reagent solution was gently mixed with 2 mL of the hydrolysate and then heated for 10 min at 100 °C using a boiling water bath. Subsequently, the samples were allowed to cool down to room temperature, and 5 mL of 95% ethanol were added. The absorbance was then recorded at a wavelength of 570 nm. A calibration curve was prepared using a leucine solution prepared daily [19]. The DH was calculated using the equation established by Adler-Nissen et al. [20]:

(1) $$DH\left(\%\right)=\frac{h}{h_{tot}}\times 100$$

where *h* (m_eq_/g protein) is the number of equivalent peptide bonds hydrolyzed and derived from the calibration curve generated using a daily prepared leucine solution by using the absorbance of the sample. *h_tot_* is the total amount of millimoles of individual amino acids per gram in the unhydrolyzed protein that can be evaluated from the amino acid profile.

To compare the hydrolysis rates among the different treatments, the initial hydrolysis rate was determined by analyzing the initial linear slope of the degree of hydrolysis curves.

Total protein concentration of the hydrolysates was estimated by Lowry’s assay [21]. A calibration curve was constructed using bovine serum albumin. The absorbance readings for both the samples and the standards were recorded at 750 nm using a Jasco V-750 spectrophotometer (Madrid, España). 

The total hydrolysis yield was determined as the ratio of the weight of the freeze-dried hydrolysate to the initial weight of the raw material:

(2) $$Hydrolysis\;yield\left(\%\right)=\frac{Weight\;of\;freeze\;dried\;hydrolysate\;(g)}{Weight\;of\;raw\;material\;(g)}\times 100$$

### 2.7. pH and Browning Intensity Measurement

The pH levels of protein hydrolysates were read at room temperature using a GLP 21 pH meter (Crison Instruments S.A., Barcelona, España). Technical buffer solutions of pH 4.00, 7.00, and 10.00 were used to calibrate the equipment before the measurements.

The browning intensity of protein hydrolysates were determined following a method described by Laroque et al. [22] with some modifications. Briefly, protein hydrolysates were diluted 10 times using distilled water, and the absorbance was measured at 420 nm using a Jasco V-750 spectrophotometer (Madrid, España).

### 2.8. Determination of Antioxidant Capacity 

#### 2.8.1. Determination of DPPH Radical-Scavenging Capacity

The DPPH radical scavenging activity was assessed following the method described by Centenaro et al. [23], with a slight modification. DPPH solution was prepared by dissolving 1 mg of DPPH (50.7 µM) in 50 mL of 95% methanol, and then placing the mixture in the dark at room temperature for 4 h before use. Subsequently, 20 µL of the liquid hydrolysate were combined with 980 µL of DPPH solution. In the control experiment, the sample was substituted with 20 µL of methanol. The mixture was vigorously shaken and left to stand for 60 min, after which the absorbance of both the sample and control solutions were measured at 517 nm using a Jasco V-750 spectrophotometer (Madrid, España). A calibration curve was prepared using Trolox. The antioxidant capacity of the protein hydrolysates was expressed as micromoles of Trolox equivalents per gram of LB muscle (µmol TE/g LB).

#### 2.8.2. Determination of ABTS Radical-Scavenging Capacity

The ABTS radical cation (ABTS^•+^) decolorization assay, as outlined by Re et al. [24], was carried out. ABTS^•+^ was generated by mixing an equal volume of ABTS stock solution with 2.45 mM potassium persulfate, and placing the mixture in the dark at room temperature for 16 h before use. The resulting ABTS^•+^ solution was diluted with ultrapure water to achieve an absorbance of approximately 0.70 at 734 nm. Subsequently, 20 μL of the diluted sample solution were introduced to 980 μL of the ABTS^•+^ reagent, and the absorbance was measured via a Jasco V-750 spectrophotometry (Madrid, España) after 20 min incubation in darkness. In the case of the blank solution, the sample was replaced with 20 µL of ultrapure water. The difference between the absorbance of the sample and the blank was referenced to a calibration curve, which was established using Trolox as a standard. The antioxidant capacity of the protein hydrolysate was expressed as micromoles of Trolox equivalents per gram of LB (µmol TE/g LB).

#### 2.8.3. Determination of Ferric-Reducing Antioxidant Power (FRAP)

The reducing capacity of the hydrolysates was estimated by the ferric reducing antioxidant power (FRAP) method following the approach designated by Benzie and Strain [25]. The FRAP reagent was freshly prepared by mixing 25 mL of sodium acetate buffer (pH 3.6), 2.5 mL of 20 mM FeCl_3_, 2.5 mL of TPTZ (2,4,6-tris.2-pyridyl-s-triazine), and 3 mL of ultrapure water. Subsequently, 30 µL of sample were mixed with 970 µL of FRAP reagent and left for 30 min at 37 °C in a water bath. The absorbance was measured at 593 nm. For the control solution, the sample was replaced with 30 µL of ultrapure water. The calibration curve was prepared using iron (II) sulfate as standard under the same conditions as the samples and results were expressed as μmol of FeSO_4_ per g of LB, μmol Fe^2+^/g LB).

### 2.9. Color Profile Determination

The color characteristics of the protein hydrolysates were assessed using a spectrophotometer (CM-2600d, Japan). The L*, a*, and b* values indicate brightness, red to green, and yellow to blue hues, respectively. These parameters were determined according to the procedure outlined by Alahmad et al. [26]. The measurements were conducted under illuminant D65 (daylight source) and observed by a standard 10° observer, as per the recommendations of the CIE (International Commission on Illumination).

### 2.10. Statistical Analysis

The results were analyzed by ANOVA (one-way) and Fisher’s Least Significant Difference (LSD) at a *p*-value < 0.05 using the R statistical package (version 19.0). The results are expressed as the mean ± standard deviation of at least in duplicates.

## 3. Results and Discussion

### 3.1. Chemical Composition of the Raw Material

The freeze-dried LB muscle had a moisture content of 3.00 ± 0.01% (*w*/*w*). Table 1 provides the proximate and elemental compositions of the freeze-dried LB muscle in dry basis. The LB muscle exhibited protein, ash, and crude fat contents of 71.9 ± 0.7%, 9.5 ± 0.2%, and 14.7 ± 0.2%, respectively, with total identified compounds accounting for 96 ± 1% on a dry basis. Carbohydrate analysis was omitted from this study due to the typically low carbohydrate content in fish, as noted by Ahmed et al. [27]. Typically, carbohydrates are often overlooked during the analysis of the proximate composition of fish. Ahmed et al. [27] highlighted that fish primarily comprise 66–81% water, 16–21% protein, 1.2–1.5% mineral, 0.2–25% fat, and less than 0.5% carbohydrate (representing less than 2.8% on a dry basis). According to this literature, the variance in composition totaling up to 100% could be attributed to carbohydrate content, as well as the precision and accuracy of methods employed for determining, protein, fat, and ash content. 

The protein content’s average value was slightly below the reported content (77.8% *w*/*w*, dry basis) for *Labeobarbus intermedius* by Geremew et al. [28]. However, both the crude fat and ash contents were higher compared to the values reported by the same authors, where the fat and ash contents were 12% and 5.1% (*w*/*w*) on a dry basis, respectively. 

Crude protein content was determined using the nitrogen content and the corresponding conversion factor derived from the amino acid profile of the LB muscle (Table 2), following the NREL standard protocols. An N-factor of 5.5 was established for LB muscle, value consistent with the recent finding by Barea et al. [10] for fish meal, which had an N-factor of 5.0, and aligning with the average value of 5.6 suggested by Mariotti et al. [29] for different classes of protein sources, including fish.. This suggests the presence of other nitrogen-containing compounds besides proteins in fish products. 

The LB muscle exhibited a comprehensive range of amino acids, with a total amino acid (TAA) content of 863 ± 22 mg/g_prot_ and a total essential amino acid (TEAA) content of 346 ± 11 mg/g_prot_ (see Table 2). The TEAA content in LB muscle was higher compared to the Common Carp (*Cyprinus carpio* L.) muscle, which had a content of 278 mg/g_prot_ [30].

Glutamic acid emerged as the predominant amino acid in LB muscle, with a content of 141.3 ± 0.1 mg/g_prot_. Additionally, LB muscle exhibited prominent levels of aspartic acid (119 ± 4 mg/g_prot_), alanine (76 ± 1 mg/g_prot_), leucine (74.0 ± 0.2 mg/g_prot_), lysine (71 ± 3 mg/g_prot_), and glycine (63.6 ± 0.4 mg/g_prot_). However, it contained relatively low amounts of cysteine (1.8 ± 0.2 mg/g_prot_) and tryptophan (5.5 ± 0.4 mg/g_prot_). Shahidi et al. [31] also reported similar findings for Capelin (*Mallotus villosus*), which exhibited high levels of glutamic acid, aspartic acid, alanine, and leucine, but lower levels of cysteine and tryptophan.

### 3.2. Degree of Hydrolysis (DH)

The degree of hydrolysis (DH) is a measure of the proportion of peptide bonds that undergo cleavage during the hydrolysis process [32]. It indicates the extent to which a protein source has been broken down, reflecting the number of cleaved peptide bonds. Therefore, the degree of peptide bond cleavage emerges as a crucial parameter in SCW hydrolysis, since it is significantly associated with protein recovery yield, biological activities, and the functional properties of the resulting protein hydrolysates [2]. 

**Figure 1:**
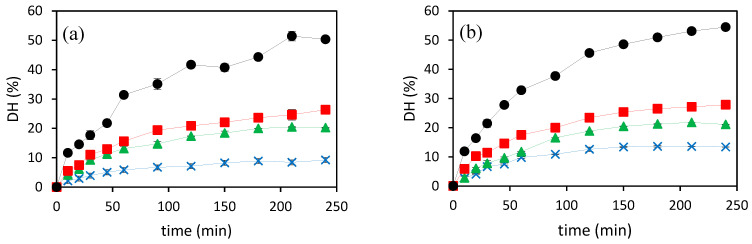
Degree of hydrolysis kinetics of SCW hydrolysates prepared by using two different pressurization agents, (**a**) N_2_ and (**b**) CO_2_, at different temperatures (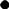 200 °C, 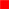 180 °C, 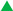 160 °C, and 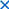 140 °C).

Figure 1 shows the DH curves of SCW hydrolysates obtained at different hydrolysis temperatures for 4 h using N_2_ and CO_2_ as pressurizing agents. DH was evaluated according to Equation (1), with a *h_tot_* value of seven according to the muscle amino acid profile. For both pressurization agents, the peptide bonds in the parent protein are broken, resulting in an increase in the release of primary amines as determined by the ninhydrin assay, corresponding to an increase in degree of hydrolysis [10].

The rate of hydrolysis increased rapidly in the first 60 min of treatment, reaching a plateau at longer treatment times at working temperatures in the range from 140 to 180 ºC. At the highest temperature considered in this work, 200 °C, a continuous increase in DH was observed in the treatment time covered in this study. The initial rate of hydrolysis, evaluated using the initial linear slope of hydrolysis curves, increased significantly with the increasing temperature for both pressurizing agents (Table 3). The DH also increased when the working temperature increased, with the highest DH observed at 200 °C, 50.3 ± 0.2% and 54.5 ± 0.4%, for N_2_ and CO_2,_ respectively. This behavior can be attributed to an increase in the ionic product, K_w_, due to higher concentration of H_3_O^+^ and OH^−^ in the medium that facilitates the release of amino groups [10].

The pressurization agent had also a significant effect on the initial hydrolysis rate and final DH, with significantly higher initial slopes for CO_2_ than for N_2_ for all temperatures assayed except at 160 °C, at which both pressurizing agents had statistically similar initial hydrolysis rate. The addition of carbon dioxide to the subcritical water medium alters the pH and the chemical environment of the reaction. Carbon dioxide reacts with water to form carbonic acid, which lowers the pH of the solution. A lower pH increases the DH, as acidic conditions generally promote hydrolytic reactions [15].

### 3.3. Total Protein Content and Hydrolysis Yield of SCW Hydrolysates

**Figure 2:**
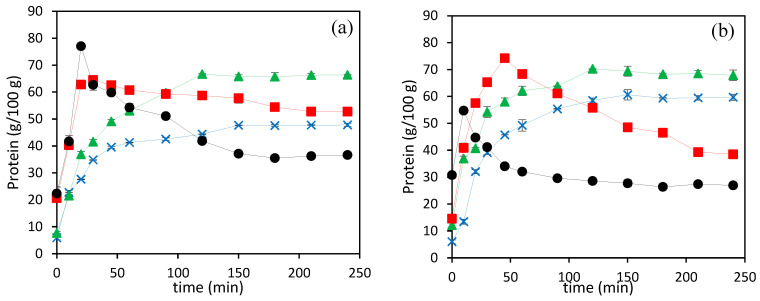
Evolution of the total protein content in SCW hydrolysates prepared by using N_2_ (**a**) and CO_2_ (**b**) at different temperatures (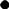 200 °C, 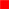 180 °C, 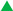 160 °C, and 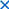 140 °C).

Figure 2a,b show the total protein content of hydrolysates obtained at different hydrolysis temperatures for 4 h using N_2_ and CO_2_ as pressurizing agents. The total protein content of hydrolysates obtained at 140 and 160 °C increased with time and reached its maximum value after 90–120 min, for both pressurizing agents. However, at 180 and 200 °C a maximum was reached in the early stages of the treatment (at 40 and 10 min, respectively), whereas further increasing the treatment time resulted in a significant reduction in the total protein content, according to the Lowry method used in this study. It is also interesting to observe that at the highest temperatures (180 and 200 °C), the protein content in the hydrolysates obtained using N_2_ as a pressurizing agent was significantly higher than the protein content in hydrolysates using CO_2_, which had significantly higher free amino acids (see Table 2). 

Asaduzzaman and Chun [11] reported that the protein content in subcritical water hydrolysates of thermal dried squid muscle decreased with increasing temperature from 160 to 280 °C, as determined by the Lowry assay, and suggested that the protein molecules decomposed to a water soluble low molecular weight organic compound at a higher temperature. In the present work, in the temperature range covered, the ionic product increases, thus facilitating the hydrolysis of complex matrixes. This was further supported by the previous results about DH, which showed that an increase in hydrolysis temperature resulted in higher DH, hydrolysis yield and total free amino acids content of hydrolysates, as is shown in Section 3.4 (see Figure 1 and Table 2 and Table 4). Therefore, the reduction in total protein content observed in this study could be also related to the Lowry assay response, which was used in this study to quantify the protein content. Barea et al. [10] determined the effect of the molecular size of proteins and pure amino acids on the response of the Lowry assay and observed that, in general, lower molecular weight proteins (<40 kDa) and free amino acids, except tyrosine and tryptophan, had a lower response to the Lowry assay due to the limitation in color formation with Lowry reagents. 

Table 4 shows the total hydrolysis yield evaluated according to Equation (2). The total hydrolysis yields increased, from 41.6 to 75.2% for N_2_ and 42.3 to 81.3% for CO_2_, by increasing the temperature from 140 to 200 °C. The increase in hydrolysates yield with temperature during sub-critical water hydrolysis can be explained by the changes in water properties [6]. 

### 3.4. Free Amino Acid Profile in the SCW Hydrolysates

The free amino acid profile of the SCW hydrolysates obtained at different hydrolysis temperatures and pressurization agents is presented in Table 2. The total free amino acid content in SCW hydrolysates obtained using N_2_ and CO_2_ increased significantly from 19 ± 1 to 143 ± 2 and 30 ± 1 to 210 ± 1 mg/g_prot_, respectively, with increasing temperature. The increase in working temperature accelerates hydrolysis due to an increase in the ionic product resulting in an increase in the production of free amino acids [13]. 

Different studies reported that degradation of amino acids could occur at certain time–temperature conditions, due to the decomposition of amino acids to organic acids and other volatile products [5,33]. Melgosa et al. [34] reported that the total free amino acid content in SCW hydrolysates from sardine waste, hydrolyzed for 6 h, increased with a temperature of up to 140 °C and then decreased in the range 190–250 °C. However, in the present study, the maximum content of total free amino acid was observed at the highest working temperature. When working with SCW, an important factor to consider is the severity factor (*R_o_*), which takes into account both the hydrolysis time and temperature, as described by the following equation [18]:

(3) $${logR}_{o}=\log\left(\;t.\exp\left(\frac{\left(T-T_{ref}\right)}{14.75}\right)\right)$$

where *t* is the treatment time (min), *T* is the operating temperature (°C) and *T_ref_* is equal to 100 °C. The maximum production of total free amino acids in this study was observed at the highest severity factor of 5.3 (at the hydrolysis temperature of 200 °C and time of 240 min), which is still lower than the severity factor (5.9–6.2) reported for achieving the maximum free amino acids from different protein sources [14,35].

Although free amino acid profiles in hydrolysates prepared using both pressurization agents showed similar trends regarding temperature, the total free amino acid contents in CO_2_ were significantly higher than in N_2_ at all hydrolysis temperatures. The addition of CO_2_ leads to a further reduction in the pH value of the hydrolysis medium, due to the solubilization of CO_2_ in the medium that increases the concentration of hydroniums in the hydrolysis medium, which promotes the release of free amino acids [14]. Barea et al. [10] also reported that addition of CO_2_ resulted in the production of 25% more free amino acids than the addition of N_2_, as pressurization agents in the hydrolysis of fish meal at 180 °C. In any case, the total free amino acid content reported by Barea et al. [10] was higher than the values reported in this work, which could be attributed to the absence of mass transfer limitations since the starting raw material was the water-soluble protein fraction of tuna fish meal, instead of a heterogeneous initial solid/liquid extraction/hydrolysis medium of the present study.

Table 2 also presents individual free amino acid content in SCW hydrolysates obtained using N_2_ and CO_2_ at different temperatures. Alanine and glycine were the most dominant amino acids found in all hydrolysates, with the highest contents, 48.31 ± 0.01 and 41.1 ± 0.01 mg/g_prot_, respectively, obtained at 200 °C using CO_2_. Barea et al. [10] also reported that alanine and glycine were the most abundant free amino acids in SCW hydrolysates of tuna fish meal. As can be seen in Table 2, the amount of alanine, glycine, valine, leucine, isoleucine, proline, methionine, phenylalanine, lysine, and tyrosine increased with temperature, whereas the content of serine and aspartic acid increased up to 180 °C, and then decreased when the temperature increased to 200 °C. The increase in the content of these amino acids as temperature increased could be attributed to the breakdown of larger protein structures into smaller peptides and amino acids, leading to an increase in the concentration of individual amino acids, including serine and aspartic acid. However, the decline of serine and aspartic acid at 200 °C might be attributed to the degradation or decomposition of these amino acids at higher temperatures. On the other hand, the highest content of threonine was observed at 160 °C but declinedwhen the temperature increased to 180 °C, eventually becoming undetectable even at the highest working temperature (200 °C). Threonine is a thermosensitive essential amino acid that was also not detected in the SCW hydrolysate from Comb penshell viscera treated at 230 °C for 15 min [7]. Even though all the above amino acids showed similar trends for both pressurizing agents, the highest content of most individual amino acids was observed in hydrolysates obtained in the presence of CO_2_. 

The total free amino acid yield was estimated as the ratio of the sum of the individual free amino acids in the hydrolysates to the sum of the total amino acid bound in the protein of the LB muscle, which has also been included in Table 2. According to the amount of free amino acid released, the yield of total free amino acids increased significantly with temperature and in the presence of CO_2_, with values ranging from 2.2 ± 0.1 to 16.6 ± 0.2 and 3.5 ± 0.1 to 24.5 ± 0.1, for hydrolysates obtained using N_2_ and CO_2_, respectively. The trend of these values was similar to the one observed by Barea et al. [10], who reported an increase in the total amino acid yield of SCW hydrolysates of fish meal with temperature and in the presence of CO_2_ as pressurization agent. 

**Figure 3:**
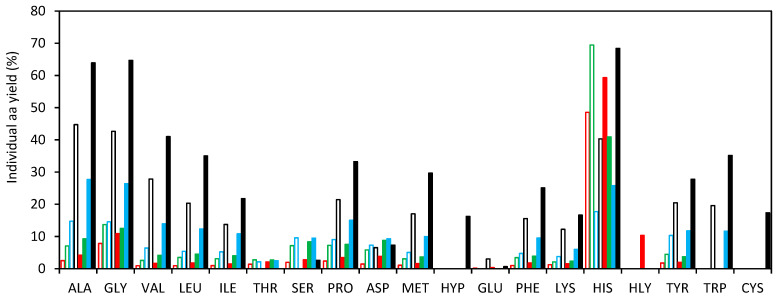
Ratio of individual amino acid content in the hydrolysate and in the LB muscle obtained by SCW with different pressurization agents and temperatures. (140 °C: 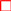 N_2_, 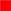 CO_2_) (160 °C: 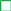 N_2_, 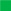 CO_2_) (180 °C: 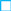 N_2_, 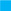 CO_2_) (200 °C: 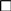 N_2_, 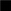 CO_2_).

Figure 3 shows the ratio for individual amino acids (“individual amino acid yield”) evaluated as the ratio of the amount of the individual free amino acid in the SCW hydrolysate to the amount of the individual amino acid bound in protein of the LB muscle. As a general trend, an increasing yield was observed with increasing temperature, according to the SCW properties in the operating temperature range. Histidine showed the highest ratio, although there was not a distinct trend observed with temperature, unlike the other amino acids. Furthermore, at the highest temperature tested in this study for both pressurization agents, histidine comprised less than 3% of the total free amino acid content. Meanwhile, the combined total of alanine, glycine, valine, and leucine constituted over 60% of the total free amino acids released in the hydrolytic medium, making them the most abundant amino acids in the hydrolysates.

### 3.5. Color, Browning Intensity and pH of Hydrolysates

Color is an important parameter of products to be used as food ingredients [5]. The color of SCW hydrolysates varied significantly with temperature and pressurizing agents (Table 4). Specifically, the lightness (L*) of the hydrolysates decreased from 37 ± 2 to 18.3 ± 0.2 for N_2_ and from 38 ± 3 to 19 ± 2 for CO_2_ as temperature increased. Similarly, the yellowness/blueness (b*) value decreased from 8.7 ± 1.3 to 3.5 ± 0.3 for N_2_ and from 9.5 ± 1.3 to 6.6 ± 0.7 for CO_2_. However, the redness/greenness (a*) value increased with rising temperature for both pressurizing agents, ranging from −0.4 ± 0.1 to 3.3 ± 0.3 for N_2_ and from 0.03 ± 0.11 to 3.1 ± 0.3 for CO_2_. The lowest lightness and the highest redness values were obtained at the highest temperature used in this study, indicating that darker hydrolysates were produced at high temperature, which might be due to the formation of the Maillard reaction products [33]. 

During heating, sugars and amino acids often undergo a series of subsequent and parallel complex reactions leading to the formation of Maillard reaction products, such as melanoidins [36]. Carbohydrate analysis was not caried out in this work since fish generally contains very low levels of carbohydrates compared to other food sources. However, a small carbohydrate content can be expected, since the sum of the protein, lipid and ash contents were less than 100%. In a recent review, Ahmed et al. [27] reported that fish contains less than 0.5% carbohydrate (<2.8% in dry base). Moreover, Muramoto et al. [37] analyzed the reducing sugar content in different fish muscles and reported that glucose and ribose are major reducing sugars found in fish muscle. Burt [38] also reported that glucose and ribose can contribute appreciably to a browning reaction.

**Figure 4:**
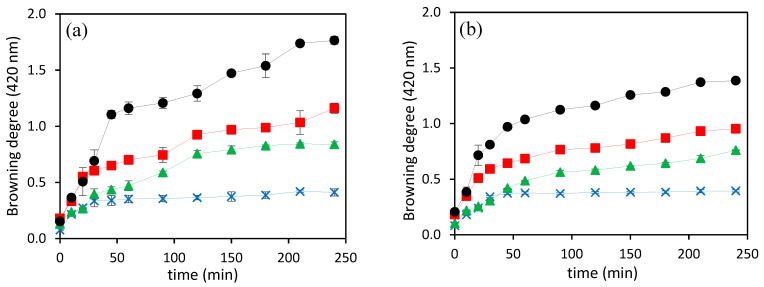
The intensity of browning in SCW hydrolysates prepared by using (**a**) N_2_ and (**b**) CO_2_ at different temperatures (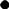 200 °C, 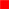 180 °C, 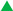 160 °C, and 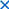 140 °C).

As can be seen in Figure 4, the intensity of browning, measured as the absorbance at 420 nm in the hydrolysates obtained using both pressurizing agents, increased alongside treatment time with increasing temperature, which resulted in darker hydrolysates. The highest browning intensities were obtained for N_2_ at all the temperatures assayed, but the difference was much higher at 200 °C. The highest absorbance value, 1.76 ± 0.03, was observed from the hydrolysate prepared at 200 °C using N_2_, which was significantly higher than the value of 1.39 ± 0.01, obtained for the hydrolysate prepared using CO_2_ at a similar working temperature. Geng et al. [39] studied the relationship between pH and the browning intensity of dried Japanese common squid, and reported that the Maillard reaction was notably suppressed at pH 4.0–6.0 and accelerated at pH 7.5–9.0. Therefore, the higher browning intensity for the hydrolysate obtained using N_2_ at 200 °C could be due to the higher pH value, 9.1 ± 0.1.

The pH of the hydrolysates significantly increased with increasing temperature ranging from 6.69 ± 0.02 to 9.1 ± 0.1 and 6.68 ± 0.04 to 7.6 ± 0.1 for N_2_ and CO_2_, respectively (Table 4). pH values were higher for the hydrolysates obtained using N_2_, especially at 200 °C. This might be due to the degradation of organic acids and other acidic compounds as well as the formation of salts and other alkaline substances, such as ammonia compounds [6]. 

### 3.6. Antioxidant Capacity of SCW Hydrolysates

**Figure 5:**
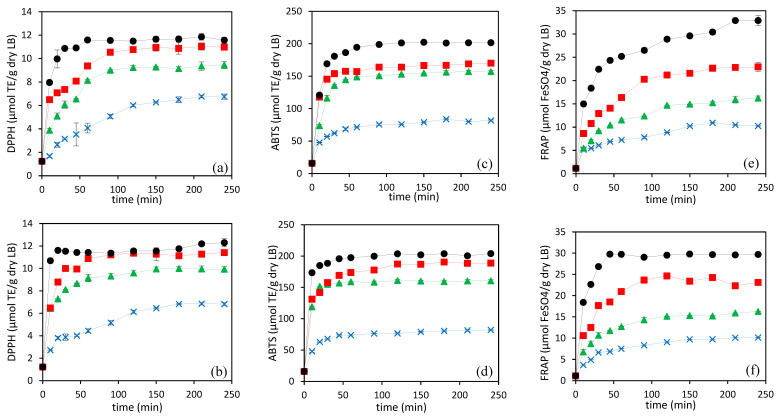
Evolution of the DPPH activity (**a**,**b**), ABTS activity (**c**,**d**), and FRAP (**e**,**f**) of SCW hydrolysates prepared by using N_2_ (**a**,**c**,**e**) and CO_2_ (**b**,**d**,**f**) at different temperatures (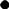 200 °C, 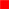 180 °C, 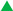 160 °C, and 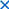 140 °C).

In the present study, LB hydrolysates showed good radical scavenging activity and reducing capacity, confirming that the hydrolysates are rich in antioxidants compounds. Figure 5 presents the antioxidant activity of SCW hydrolysates along 4 h of hydrolysis at different working temperatures, using N_2_ and CO_2_ as pressurizing agents. DPPH (Figure 5a,b), ABTS (Figure 5c,d), and FRAP (Figure 5e,f) assays were performed. The antioxidant activities of the hydrolysates increased rapidly in the first 50–60 min, reaching a plateau from treatment times of approximately 100 min. The maximum antioxidant activities of the hydrolysates increased with increasing temperature, with the highest DPPH radical scavenging activity of 12.2 ± 0.2 and 12.0 ± 0.3 µmol TE/g dry LB (16.2 ± 0.3 and 14.8 ± 0.4 µmol TE/g hydrolysate, considering the hydrolysis yield reported in Table 4), ABTS radical scavenging activity of 202 ± 1 and 204 ± 1 µmol TE/g dry LB (269 ± 1 and 251 ± 1 µmol TE/g hydrolysate, considering the hydrolysis yield reported in Table 4), and FRAP of 33±1 and 29.7 ± 0.1 μm FeSO_4_/g dry LB (44 ± 1 and 36.5 ± 0.1 µmol TE/g hydrolysate, considering the hydrolysis yield reported in Table 4) observed at 200 °C using N_2_ and CO_2_, respectively. During hydrolysis, several low molecular weight peptides and free amino acids are generated depending on temperature and time, which are supposed to be responsible for the free-radical scavenging activity and reducing capacity of the hydrolysates [6]. 

Some studies have shown that the DH was not directly correlated with the antioxidant capacity of the hydrolysates [5,40]. However, in this study, the antioxidant capacity of the hydrolysates obtained at different time intervals and working temperatures, using both pressurizing agents, showed a similar trend as the DH curves (see Figure 2 and Figure 5). The correlation coefficients for both pressurizing agents were determined according to the Pearson product moment correlation, to observe the relationship between antioxidant capacities and DH. A statistically significant correlation at the 95.0% confidence level, and positive correlation coefficients between antioxidant activities and DH were observed, with values of correlation coefficients of 0.85 and 0.77 for DPPH radical scavenging activity, 0.84 and 0.75 for ABTS radical scavenging activity, and 0.96 and 0.89 for reducing capacity in the presence of N_2_ and CO_2_, respectively (n = 48, the number of pairs of data values used to compute each coefficient). 

Apart from the ABTS scavenging activity of hydrolysate prepared at 180 °C, and the reducing capacity of hydrolysates obtained at 200 °C, there were no significant differences observed in the effects of the pressurizing agents on antioxidant activity at the final hydrolysis time across the range of temperatures tested, when expressed per g of LB. The hydrolysate obtained at 180 °C using CO_2_ showed higher ABTS radical activity than the hydrolysate obtained using N_2_. This may be due to the higher content of free amino acids in the hydrolysate prepared at 180 °C using CO_2_. However, the same trend was not observed at 200 °C where both pressurizing agents showed similar ABTS radical scavenging, while the free amino acid content in the hydrolysate obtained at 200 °C using CO_2_ was higher than N_2_. 

Moreover, the hydrolysate obtained at 200 °C using N_2_ had a higher reducing capacity than CO_2_, which had a significantly higher DH value and free amino acid content. This suggests that, in addition to smaller peptides and free amino acids, other chemicals produced by SCW hydrolysis at high temperatures also play a significant role in the reducing and radical scavenging capacity of hydrolysates. Melanoidins, brown-colored compounds, can act as antioxidants [36]. As can be seen in Figure 4, the hydrolysates prepared at 200 °C for 4 h using N_2_ had significantly higher browning intensity than hydrolysate obtained using CO_2_. Therefore, the higher reducing capacity of the hydrolysate prepared at the final hydrolysis time and highest temperature in the presence of N_2_ might be due to the higher concentration of Maillard reaction products and other compounds formed during the decomposition of organic acids.

### 3.7. Conclusions

The hydrolysis of low-valued ray-finned fish (*Labeobarbus nedgia*) was examined using subcritical water (SCW), employing nitrogen (N_2_) and carbon dioxide (CO_2_) as pressurizing agents within a temperature range of 140 to 200 °C. The degree of hydrolysis, free amino acid content, antioxidant activity, and browning intensity of the hydrolysates increased with increasing working temperature from 140 to 200 °C for both pressurizing gases. Using CO_2_ as the pressurizing agent increased the DH and made the production of free amino acids more efficient. However, the protein content decreased when increasing the temperature beyond 160 °C, due to the lower response of Lowry analysis to smaller peptides and free amino acids generated in the hydrolysis process. Therefore, in studies involving the hydrolysis processes at elevated temperatures, alternative methods for protein determination, particularly those capable of accurately quantifying smaller peptides and free amino acids, should be considered to ensure the reliability and accuracy of the results. 

## Figures and Tables

**Table 1:** Proximate and elemental composition of the freeze-dried LB muscle in dry basis.

Compound	Composition, % (*w*/*w*)	Element	Composition, % (*w*/*w*)
Proteins	71.9 ± 0.7	C	51 ± 1
Lipids	14.7 ± 0.2	H	8.1 ± 0.1
Ash	9.5 ± 0.2	N	13.1 ± 0.1
		O	18 ± 2
		S	0.6 ± 0.1

**Table 2:** Amino acid profile of freeze-dried LB muscle and free amino acid (FAA) profile of protein hydrolysates obtained using different pressurizing agents at different temperatures.

		Soluble Free Amino Acid (mg/g_prot_)							
		SCW Hydrolysis with N_2_				SCW Hydrolysis with CO_2_			
AA	LB Muscle (mg/g_prot_)	140 °C	160 °C	180 °C	200 °C	140 °C	160 °C	180 °C	200 °C
ALA	76 ± 1	1.88 ± 0.03 ^h^	5.35 ± 0.02 ^f^	11.1 ± 0.1 ^d^	33.8 ± 0.3 ^b^	3.21 ± 0.04 ^g^	7.01 ± 0.04 ^e^	20.93 ± 0.03 ^c^	48.31 ± 0.01 ^a^
GLY	63.6 ± 0.4	4.98 ± 0.03 ^i^	8.7 ± 0.04 ^e^	9.2 ± 0.1 ^d^	27.1 ± 0.2 ^b^	7.0 ± 0.1 ^g^	8.0 ± 0.1 ^f^	16.77 ± 0.02 ^c^	41.1 ± 0.01 ^a^
VAL	37.1 ± 0.2	0.34 ± 0.03 ^h^	1.0 ± 0.1 ^f^	2.4 ± 0.1 ^d^	10.3 ± 0.1 ^b^	0.62 ± 0.01 ^g^	1.54 ± 0.01 ^e^	5.17 ± 0.01 ^c^	15.21 ± 0.04 ^a^
LEU	74.0 ± 0.2	0.68 ± 0.04 ^i^	2.59 ± 0.01 ^f^	4.0 ± 0.1 ^d^	15.0 ± 0.2 ^b^	1.31 ± 0.03 ^h^	3.34 ± 0.04 ^e^	9.14 ± 0.03 ^c^	25.93 ± 0.01 ^a^
ILE	30.8 ± 0.2	0.3 ± 0.1 ^h^	1.0 ± 0.1 ^f^	1.61 ± 0.02 ^d^	4.2 ± 0.1 ^b^	0.49 ± 0.04 ^g^	1.24 ± 0.01 ^e^	3.33 ± 0.03 ^c^	6.69 ± 0.02 ^a^
THR	44 ± 2	0.6 ± 0.1 ^e^	1.2 ± 0.01 ^b^	0.93 ± 0.02 ^d^	N. D.	0.94 ± 0.04 ^d^	1.18 ± 0.03 ^b^	1.08 ± 0.01 ^c^	N. D.
SER	36 ± 2	0.7 ± 0.1 ^g^	2.6 ± 0.1 ^c^	3.5 ± 0.1 ^a^	N. D.	1.1 ± 0.1 ^e^	3.05 ± 0.01 ^b^	3.45 ± 0.04 ^a^	0.95 ± 0.01 ^f^
PRO	35 ± 1	0.82 ± 0.03 ^h^	2.57 ± 0.02 ^f^	3.21 ± 0.03 ^d^	7.6 ± 0.1 ^b^	1.24 ± 0.03 ^g^	2.68 ± 0.01 ^e^	5.33 ± 0.03 ^c^	11.77 ± 0.03 ^a^
ASP	119 ± 4	1.71 ± 0.02 ^g^	6.94 ± 0.02 ^e^	8.68 ± 0.03 ^c^	7.8 ± 0.1 ^d^	4.55 ± 0.03 ^f^	10.5 ± 0.2 ^b^	11.1 ± 0.1 ^a^	8.68 ± 0.01 ^c^
MET	33 ± 1	0.35 ± 0.01 ^h^	1.01 ± 0.03 ^f^	1.7 ± 0.1 ^d^	5.6 ± 0.1 ^b^	0.53 ± 0.03 ^g^	1.20 ± 0.01 ^e^	3.27 ± 0.03 ^c^	9.72 ± 0.01 ^a^
HYP	5 ± 1	N. D.	N. D.	N. D.	N. D.	N. D.	N. D.	N. D.	0.77 ± 0.01 ^a^
GLU	141.3 ± 0.1	0.24 ± 0.04 ^de^	N. D.	N. D.	4.3 ± 0.1 ^a^	0.5 ± 0.1 ^d^	N. D.	N. D.	0.9 ± 0.2 ^c^
PHE	42 ± 2	0.42 ± 0.02 ^i^	1.45 ± 0.03 ^f^	2.0±0.1 ^d^	6.5 ± 0.1 ^b^	0.75 ± 0.01 ^h^	1.63 ± 0.01 ^e^	4.01 ± 0.02 ^c^	10.55 ± 0.03 ^a^
LYS	71 ± 3	0.88 ± 0.01 ^h^	1.48 ± 0.01 ^gh^	2.7 ± 0.1 ^e^	8.7 ± 0.2 ^b^	1.10 ± 0.04 ^gh^	1.66 ± 0.02 ^fg^	4.27 ± 0.03 ^d^	11.8 ± 0.3 ^a^
HIS	9 ± 3	4.4 ± 0.1 ^f^	6.31 ± 0.03 ^b^	1.61 ± 0.04 ^i^	3.66 ± 0.03 ^g^	5.4 ± 0.1 ^e^	3.72 ± 0.01 ^g^	2.34 ± 0.01 ^h^	6.21 ± 0.01 ^c^
HLY	5.3 ± 0.4	N. D.	N. D.	N. D.	N. D.	0.5 ± 0.1 ^b^	N. D.	N. D.	N. D.
TYR	33.5 ± 0.2	0.59 ± 0.01 ^h^	1.49 ± 0.02 ^e^	3.5 ± 0.1 ^d^	6.9 ± 0.2 ^b^	0.66 ± 0.02 ^h^	1.24 ± 0.02 ^f^	3.9 ± 0.1 ^c^	9.31 ± 0.01 ^a^
TRP	5.5 ± 0.4	N. D.	N. D.	N. D.	1.07 ± 0.01 ^b^	N. D.	N. D.	0.64 ± 0.04 ^c^	1.92± 0.01 ^a^
CYS	1.8 ± 0.2	N. D.	N. D.	N. D.	N. D.	N. D.	N. D.	N. D.	0.32 ± 0.02 ^c^
TAA or TFAA	863 ± 22	19 ±1 ^j^	43.6 ± 0.4 ^f^	56 ± 1 ^d^	143 ± 2 ^b^	30 ± 1 ^i^	48 ± 1 ^e^	95 ± 1 ^c^	210 ± 1 ^a^
Yield (%)		2.2 ± 0.1 ^j^	5.1 ± 0.1 ^f^	6.5 ± 0.1 ^d^	16.6 ± 0.2 ^b^	3.5 ± 0.1 ^i^	5.6 ± 0.1 ^e^	11.0 ± 0.1 ^c^	24.5 ± 0.1 ^a^
TEAA	346 ± 11	8.6 ± 0.3 ^g^	17.4 ± 0.3 ^e^	20 ± 1 ^d^	61 ± 1 ^b^	11.7 ± 0.3 ^f^	16.8 ± 0.2 ^e^	36.6 ± 0.2 ^c^	95.4 ± 0.4 ^a^

Values with different letters in each row are significantly different when applying the Fisher’s Least Significant Difference (LSD) method at *p*-value < 0.05. TAA, total amino acid; TEAA, total essential amino acid; and N. D., not detected. Yield of total free amino acid, Yield (%) = TFAA/TAA × 100; TFAA, total free amino acid in hydrolysates.

**Table 3:** Initial hydrolysis rate at different temperatures for N_2_ and CO_2_ as pressurization agents.

Temperature (°C)	Pressurizing Agent	
	N_2_	CO_2_
140	0.12370 ± 0.00004 ^d B^	0.188 ± 0.005 ^d A^
160	0.276 ± 0.005 ^c A^	0.27 ± 0.02 ^c A^
180	0.38 ± 0.02 ^b B^	0.43 ± 0.02 ^b A^
200	0.541 ± 0.007 ^a B^	0.61 ± 0.01 ^a A^

Values with different capital letters in each row indicate significant differences between the slopes for pressurizing agents and values with different lowercase letters in column indicate significant differences among the slopes for the different temperatures when applying the Fisher’s Least Significant Difference (LSD) method at *p*-value < 0.05.

**Table 4:** Color, pH, and hydrolysis yield of the SCW hydrolysates obtained at various temperatures and pressurizing agents.

Pressurization Agents	Temperature (°C)	L*	a*	b*	pH	Hydrolysis Yield (%)
N_2_	140	37 ± 2 ^a^	−0.4 ± 0.1 ^d^	8.7 ± 1.3 ^ab^	6.69 ± 0.02 ^f^	41.6
	160	23 ± 2 ^b^	1.6 ± 0.5 ^c^	8.7 ± 0.2 ^ab^	7.0 ± 0.1 ^d^	52.5
	180	20 ± 1 ^bc^	2.7 ± 0.5 ^ab^	8.6 ± 0.9 ^abc^	7.78 ± 0.03 ^b^	66.0
	200	18.3 ± 0.2 ^c^	3.3 ± 0.3 ^a^	3.5 ± 0.3 ^d^	9.1 ± 0.1 ^a^	75.2
CO_2_	140	38 ± 3 ^a^	0.03 ± 0.11 ^d^	9.5 ± 1.3 ^a^	6.68 ± 0.04 ^f^	42.3
	160	22 ± 5 ^bc^	2.3 ± 0.4 ^bc^	7.4 ± 2.4 ^bc^	6.89 ± 0.04 ^e^	58.5
	180	20 ± 3 ^bc^	2.8 ± 0.3 ^ab^	6.5 ± 1.1 ^c^	7.3 ± 0.1 ^c^	69.4
	200	19 ± 2 ^bc^	3.1 ± 0.3 ^a^	6.6 ± 0.7 ^c^	7.6 ± 0.1 ^b^	81.3

Values with different letters in each column are significantly different when applying the Fisher’s Least Significant Difference (LSD) method at *p*-value < 0.05.

## Data Availability

The original contributions presented in the study are included in the article, further inquiries can be directed to the corresponding author.
